# Distinct functions of S-ketamine and R-ketamine in mediating biobehavioral processes of drug dependency: comments on Bonaventura et al.

**DOI:** 10.1038/s41380-022-01629-0

**Published:** 2022-05-20

**Authors:** Insop Shim

**Affiliations:** Department of Physiology, College of Medicine, Kyung Hee University, Seoul, 02447 Republic of Korea

**Keywords:** Neuroscience, Biomarkers

## To the Editor:

The N-methyl-D-aspartate (NMDA) receptor antagonist ketamine, a racemic mixture comprising equal parts of (S)-ketamine and (R)-ketamine enantiomers is known to exert rapid and sustained antidepressant effects in treatment-resistant patients with major depressive disorder or bipolar depression [1]. Ketamine has addictive potential due to its promising use as a therapeutic drug and has been widely used as a recreational drug [2]. The behavioral effects of racemic ketamine have been studied extensively in preclinical models [3], predictive of abuse liability in humans, but relatively little is known about the effects of each enantiomer on drug dependency. Ketamine and its enantiomers show distinct neurobehavioral and psychological effects that are investigated in preclinical and clinical psychiatric research [3, 4]. Their addictive potential may mostly depend on the extent of these pharmacological and psychological effects which may greatly differ between the enantiomers. Thus, Bonaventura et al. recently have addressed this issue and provided a comprehensively pharmacological and behavioral profiling of (S)-ketamine and (R)-ketamine, demonstrating that two enantiomers have distinct functions in mediating physiological processes of drug dependency [5]. I praise their significant contribution on understanding implications of two enantiomers for abuse liability, but there are several issues worthy to discuss. The differential effects of two enantiomers reported in their study call for comments and controversy [6].

The authors reported specific effects of subanesthetic doses of (S)- ketamine, but not (R)-ketamine, on increases in metabolic activity, dopamine (DA) tone, and opioid receptor signaling in the medial prefrontal cortex (mPFC). Based on (S)-ketamine’s pharmacological profile, they reasoned that the drug would exhibit higher potential liability for abuse than (R)-ketamine. To test this hypothesis, a series of behavioral studies were performed. This team examined whether two enantiomers differ in their effects on locomotor activity, psychomotor sensitization and conditioned place preference (CPP). They reported that acute injections of (S)-ketamine, but not (R)-ketamine-induced locomotor activity that was opioid receptor-dependent. Repeated (S)-ketamine injections produced marked behavioral sensitization and CPP, whereas (R)-ketamine injections resulted in a much smaller or no behavioral effects. Mice that received repeatedly (S)-ketamine injections showed significantly higher locomotor activity, compared to both (R)-ketamine- or vehicle-treated mice, indicating that they developed locomotor sensitization to (S)-ketamine. The authors concluded that like most of psychostimulant drugs, mice developed psychomotor sensitization to repeatedly injections of (S)-ketamine but not (R)-ketamine and that (S)-ketamine had significantly greater potential for abuse liability than (R)-ketamine. This seems quite a reasonable hypothesis to test behavioral effects of two enantiomers based on pharmacological profiles. However, we have some doubts about this conclusion, and it should be careful to interpret the data they presented before drawing a solid conclusion. They reported that acute injections with (S)-ketamine (5, 10 and 20 mg/kg) were more potent than (R)-ketamine in increasing locomotor activity, with (R)-ketamine increasing it only at 20 mg/kg dose. The authors did not find any pronounced increase in locomotor activity following injections of (R)-ketamine. This finding markedly contrasts the previous other reports showing that (R)-ketamine (10 to 30 mg/kg) increased robust locomotor activity in mice, producing more potent effects than (S)-ketamine (30 mg/kg) [7]. Behavioral effects of (S)- and (R)-ketamine have been not consistently observed [7]. However, the authors did not describe properly these inconsistent results in detail. Critically, it is worthy to discuss more what factors and mechanisms such as procedural differences, duration of measurement or species differences could explain the different findings in these reports.

It should be also noted that they unusually adopted a protocol whereby sensitization occurs with only during 3 days-developmental period, which was too short to evaluate behavioral sensitization produced by each enantiomer. This adopted period for drug treatment is relatively far from investigating the aspects of development and expression of behavioral sensitization, which is a form of neurobehavioral plasticity, evidenced by an enhanced locomotor response to a subsequent injection of drug [8]. In general, the rodent experiment for testing behavioral sensitization consists of three phases: a 7-day developmental phase, a 3-day withdrawal phase and one testing phase [8]. Indeed, other study reported that longer treatment with (R, S) ketamine such as five or more days produced a pronounced development of locomotor sensitization [9]. In addition, although authors stated limitation of their study, only a single dose of each enantiomer in the behavioral sensitization was utilized, showing that mice developed psychomotor sensitization to repeated injections of (S)-ketamine (20 mg/kg), but not (R)-ketamine (20 mg/kg). However, (S)-ketamine and (R)-ketamine with lower doses should be tested to clarify here their abuse liability. It has been shown that a significant neurobehavioral plasticity is evident at doses that are near or below the threshold for locomotor stimulation according to the previous research, demonstrating ketamine sensitization at very low doses [8]. A number of studies has clearly demonstrated that low doses of (R, S) ketamine (5, 10, 20 or 50 mg/kg i.p.) produced marked sensitization [10]. Importantly, increases in response following repeated administration, reflective of ketamine sensitization, occurred not only at 20 mg/kg, but also at 5 and 10 mg/kg, despite the lack of a significant stimulant response at the lower doses on day 1. Therefore, the possibility cannot be ruled out that mice may develop psychomotor sensitization to repeated injections of (R)-ketamine at lower doses and the sustained sensitizing or repeated effect of ketamine at lower dosages may be responsible for the potency of the effects on synaptic plasticity. Therefore, it is essential that more long-term treatment period and lower dosages of the drugs should be adopted to clearly assess differential effects of two enantiomers in behavioral sensitization model.

Regarding specific effects of (S)-ketamine on increases in metabolic activity, DA tone, and opioid receptor signaling in the mPFC, Positron emission tomography used in their study is relatively indirect measure to estimate the concentration of DA in the extracellular space. I would suggest that more direct methods are necessary to measure the extracellular DA release in the mPFC or basal ganglia, utilizing such as in vivo microdialysis or voltammetry method in freely moving animals in order to test whether the differential actions of (S)-ketamine and (R)-ketamine enantiomers is dependent on the central DA machinery. Additionally, ketamine, a NMDA receptor antagonist, is known to have a different mechanism of action rather than other classes of antidepressants, whereas the majority of antidepressants act by modulating the central monoaminergic neurotransmission. This fact raises the possibility that mechanisms other than NMDA receptors may be involved in (S)- or (R)-ketamine-induced sensitization, CPP and self-administrations of their findings. Although the authors emphasized the role of opioid systems on acute (S)- and (R)-ketamine-induced locomotor, they did not specify its potential role in long-term behavioral sensitization model. The authors only observed that (S)-ketamine but not (R)-ketamine acutely induced locomotor activity that was blocked by pretreatment with the opioid antagonist naltrexone. The precise role of the opioid systems as well as DA, 5-HT or GABAergic systems should be examined in mediating the development of sensitization produced by two isoforms, and the expression of the response in sensitized animals.

Considering searching targets for treatment on addiction mechanisms, there will be a great deal of interest in uncovering the neural mechanisms underlying behavioral effects of each enantiomer. Even though the authors made their valuable contributions of ketamine for abuse liability, more precise mechanisms explaining each enantiomer remained to be elucidated. These studies will be warranted to extend our understanding of addictive as well as antidepressant-like properties of ketamine.
